# Predicting Transmembrane Helix Packing Arrangements using Residue Contacts and a Force-Directed Algorithm

**DOI:** 10.1371/journal.pcbi.1000714

**Published:** 2010-03-19

**Authors:** Timothy Nugent, David T. Jones

**Affiliations:** Bioinformatics Group, Department of Computer Science, University College London, London, United Kingdom; Stockholm University, Sweden

## Abstract

Alpha-helical transmembrane proteins constitute roughly 30% of a typical genome and are involved in a wide variety of important biological processes including cell signalling, transport of membrane-impermeable molecules and cell recognition. Despite significant efforts to predict transmembrane protein topology, comparatively little attention has been directed toward developing a method to pack the helices together. Here, we present a novel approach to predict lipid exposure, residue contacts, helix-helix interactions and finally the optimal helical packing arrangement of transmembrane proteins. Using molecular dynamics data, we have trained and cross-validated a support vector machine (SVM) classifier to predict per residue lipid exposure with 69% accuracy. This information is combined with additional features to train a second SVM to predict residue contacts which are then used to determine helix-helix interaction with up to 65% accuracy under stringent cross-validation on a non-redundant test set. Our method is also able to discriminate native from decoy helical packing arrangements with up to 70% accuracy. Finally, we employ a force-directed algorithm to construct the optimal helical packing arrangement which demonstrates success for proteins containing up to 13 transmembrane helices. This software is freely available as source code from http://bioinf.cs.ucl.ac.uk/memsat/mempack/.

## Author Summary

Alpha-helical transmembrane proteins constitute a significant proportion of the proteins encoded in a typical genome and are involved in a wide variety of important biological processes. Many common diseases including diabetes, hypertension and epilepsy have been related to transmembrane protein dysfunction, therefore they represent one of the most important classes of protein for pharmaceutical intervention. However, due to the experimental difficulties of structure determination, this class of protein is severely under-represented in structural databases. Here, we present a novel approach that is able to predict lipid exposure, residue contacts, helix-helix interactions and finally the optimal helical packing arrangement of a transmembrane protein. Under stringent cross-validation, our approach demonstrates a significant improvement in prediction over existing software. This method can be used to gain insights into transmembrane protein folding and enhance the quality of *ab initio* modelling, while providing testable hypotheses for a variety of studies including protein design, mutagenesis and thermostability experiments.

## Introduction

Alpha-helical transmembrane (TM) proteins constitute roughly 30% of the proteins encoded in a typical genome and are involved in a wide variety of important biological processes including cell signalling, transport of membrane-impermeable molecules and cell recognition. Many are also prime drug targets, and it has been estimated that more than half of all drugs currently on the market target membrane proteins [1]. Despite significant efforts to predict TM protein topology [2],[3],[4], comparatively little attention has been directed toward developing a method to pack the helices together. Since the membrane-spanning region is predominantly composed of alpha-helices with a common alignment, this task should in principle be easier than predicting the fold of globular proteins as the longitudinal constraints of helix packing mostly reduces the solution space from three dimensions to two. However, topologies consisting of large numbers of TM helices as well as structural features including re-entrant, tilted and kinked helices render simple approaches that may work for regularly packed proteins unable to predict the diverse packing arrangements now present in structural databases.

Early attempts to predict TM protein folds were based on sequence similarity to proteins with a known three-dimensional structure, using statistically derived environmental preference parameters combined with experimentally determined features [5]. Another method calculated amino acid substitution tables for residues in membrane proteins where the side chain was accessible to lipid. By comparing observed substitutions obtained from sequence alignments of TM regions, accessibility of residues to the lipid could be predicted. In combination with a Fourier transform method to detect alpha-helices, the buried and exposed faces could then be discriminated and the presence of charged residues used to construct a three-dimensional model [6]. Other methods also made use of exposed surface prediction to allocate helix positions, in combination with an existing framework for globular protein structure prediction involving the combinatorial enumeration of windings over a predefined architecture followed by the selection of preferred folds [7]. However, these methods were only suitable for 7 TM helix bundles such as rhodopsin and were unsuitable for other topologies.

More recently, a modified version of the fragment-based protein tertiary structure prediction method FRAGFOLD [8] was modified to model TM proteins. FRAGFOLD is based on the assembly of super-secondary structural fragments using a simulated annealing algorithm in order to narrow the search of conformational space by pre-selecting fragments from a library of highly resolved protein structures. FILM [9] added a membrane potential to the FRAGFOLD energy terms which was derived from the statistical analysis of a data set of TM proteins with experimentally defined topologies. Results obtained by applying the method to small membrane proteins of known three-dimensional structure showed it could predict both helix topology and conformation at a reasonable accuracy level. Despite these good results, the combinatorial complexity of such *ab initio* protein folding methods means that it is unfeasible to use such approaches for large TM structures, many of which are longer than 150 residues. Modification of another globular protein *ab initio* modelling program, ROSETTA [10], added an energy function that described membrane intra-protein interactions at atomic level and membrane protein/lipid interactions implicitly, while treating hydrogen bonds explicitly [11]. Results suggest that the model captures the essential physical properties that govern the solvation and stability of TM proteins, allowing the structures of small protein domains, up to 150 residues, to be predicted successfully to a resolution of less than 2.5 Å. A recent enhancement of the algorithm demonstrated that by constraining helix-helix packing arrangements at particular positions based on local sequence-structure correlations for each helix of the interface independently, TM proteins with more complex topologies could be modelled to within 4 Å of the native structure [12].

The prediction of helix-helix interactions, derived from residue contacts and topology, has only recently been investigated in TM proteins due to the relative paucity of TM protein crystal structures. In contrast, a number of globular protein contact predictors exist based on a variety of machine learning algorithms [13],[14], and contact prediction has also been used to assess globular protein models submitted to the Critical Assessment of Structure Prediction (CASP) experiment [15]. However, analysis has shown that such globular proteins contact predictors perform poorly when applied to TM proteins, most likely due to differences between TM and globular interaction motifs [16]. A number of studies have identified structural and sequence motifs recurring frequently during helix–helix interaction in TM proteins. One investigation analysed interacting helical pairs according to their three-dimensional similarity, allowing three quarters of pairs to be grouped into one of five tightly clustered motifs [17]. The largest of these consisted of an anti-parallel motif with left-handed packing angles, stabilised by the packing of small side chains every seven residues, while right-handed parallel and anti-parallel structures showed a similar tendency though spaced at four-residue intervals. Another study identified a specific aromatic pattern, aromatic-XX-aromatic, which was demonstrated to stabilise helix-helix interactions during assembly [18], while others include the GXXXG motif found in glycophorin A [19], heptad motifs of leucine residues [20], and polar residues through formation of hydrogen bonds [21].

The discovery of these recurring motifs, and the likelihood that there are more as yet undiscovered, suggests predictability by a generalised pattern search strategy. Recently, two methods have been developed that attempt to predict residue contacts and helix-helix interaction. TMHcon [16] uses a neural network in combination with profile data, residue co-evolution information, predicted lipid exposure using the LIPS method [22], and a number of TM protein specific features, such as residue position within the TM helix, in order to predict helix-helix interaction. TMhit [23] uses a two-level hierarchical approach in combination with a support vector machine (SVM) classifier. The first level discriminates between contacts and non-contacts on a per residue basis, before the second level determines the structure of the contact map from all possible pairs of predicted contact residues therefore avoiding the high computational cost incurred by the quadratic growth of residue pair prediction.

Here, we present a novel method to predict lipid exposure, residue contacts, helix-helix interactions and finally the optimal helical packing arrangements of TM proteins. Using molecular dynamics data to label residues potentially exposed to lipid, we have trained and cross-validated a SVM classifier to predict per residue lipid exposure with 69% accuracy. This information is combined with PSI-BLAST profile data and a variety of sequence-based features to train an additional SVM to predict residue contacts. Combining these results with *a priori* topology information, we are able to predict helix-helix interaction with up to 65% accuracy under stringent cross-validation on a non-redundant test set of 74 protein chains. We then tested the ability of the method to discriminate native from decoy helical packing arrangement using a decoy set of 2811 structures. By comparing our predictions with the test set, we were able to identify the native packing arrangement with up to 70% accuracy. All these performance metrics represents significant improvements over existing methods. In order to visualise the global packing arrangement, we adopted a graph-based approach. By employing a force-directed algorithm, the method attempts to minimise edge crossing while maintaining uniform edge length, attributes common in native structures. Finally, a genetic algorithm is used to rotate helices in order to prevent residue contacts occurring across the longitudinal helix axis.

## Materials and Methods

### Data sets

For any machine learning task, the use of a high quality data set for both training and validation purposes is essential. Our data set was based on a previously described crystal structure set [4] which contained data initially collected from MPTOPO [24], OPM [25], PDB_TM [26] and SWISS-PROT [27] before fragments, sequences containing chain breaks and non-native TM proteins such as venoms and colicins were removed. OPM was used to define TM helix boundaries, although where a visual inspection appeared to indicate incorrect placement of the membrane, PDB_TM helix boundary definitions were used instead. The data set was homology reduced at the 40% sequence identity level leaving 131 sequences, of which the 74 which contained at least two TM helices were used to predict residue contacts. For 53 of these multi-spanning sequences, and a further 24 single-spanning proteins, we were able to obtain molecular dynamics data from the Course Grained Database (CGDB) [28] which was used for lipid exposure prediction. We chose not to predict interactions between TM helices and re-entrant helices, found in many channels such as Aquaporin, as they are thought to be involved in channel gating and thus move into and out of the membrane region depending on physiological conditions. Including re-entrant helices would therefore be likely to introduce noise into the data set as contacts could be both positive and negative training examples.

### Predicting lipid exposure

During TM protein crystallisation, detergents are used extensively for membrane solubilisation and then act as mimics of the lipid bilayer due to their self-assembly properties. As a result, crystallographic data rarely contains information regarding the positions of lipid molecules, therefore hindering the study, and prediction, of lipid exposed regions of TM protein. For investigating TM topology, a number of automated methods exist that attempt to position the protein within the membrane [25],[26]. However, these methods are inappropriate for accurate studies of lipid exposure as they do not take into account the solvent-filled cavities and channels found in many TM proteins. To address this, we used the CGDB, a resource of coarse-grained simulation data, which contains analysis of lipid-protein interactions following 200 ns of molecular dynamics using GROMACS [29] to randomly surround TM proteins in dipalmitoylphosphatidylcholine lipids and solvent. A snapshot of each protein in its optimum position within the bilayer and residue statistics throughout the simulation are available. While difficult to validate, the approach has proved successful in reproducing the behaviour of equivalent atomistic simulations of model proteins, as well as allowing the insertion of various test peptides whose final configurations were in agreement with experimental data [30]. Additionally, channel-containing proteins such as aquaporin and potassium channels are solvent rather than lipid filled at the end of simulation.

To train a SVM classifier, we used CGDB data to label residues that were lipid exposed. For the 77 proteins within out data set where CGDB data was available, each residue within the membrane was labelled as lipid exposed where the fraction of simulation time exposed to DPPC lipid was greater than 0.5. PSI-BLAST [31] was used to generate position-specific scoring matrices for each of the 77 proteins in the data set using the UniRef 90 database. Two iterations were performed with a profile-inclusion E-value threshold of 0.001. For each residue in a sequence, a sliding window approach was used with a window size of 7, creating a feature vector of length 140 centred on the target residue. To determine this windows size, the data set was split randomly into two and the highest scoring window which ranked equally in each split was selected, therefore demonstrating consistency between data sets and reducing the risk of overfitting. Where the window extended beyond the protein termini, empty feature values were set to zero. All values for each feature position where then normalised by Z-score to enable faster SVM convergence. In training, the target sequence, along with any other sequences with an E-value less than 1e-4, were excluded. We used SVM-Light [32] and a radial basis function kernel, in combination with a grid search of SVM parameters. Matthews Correlation Coefficient (MCC) was used to optimise these values as it has been shown to be a more robust measure than using recall or precision alone [33].

### Contact definitions

In order to make direct comparisons with other methods, we used three thresholds to consider a pair of residues to be in contact. Firstly, a maximal distance of 8 Å between their C-beta atoms (C-alpha for glycine) [13],[14] (contact definition 1). Secondly, the distance between any two atoms from an interacting pair is less than the sum of their van der Waals radii plus a threshold of 0.6 Å [23] (contact definition 2). Thirdly, the minimal distance between side chain or backbone heavy atoms in an interacting pair is less than 5.5 Å [16] (contact definition 3). We defined TM helices as interacting if one residue from each helix was observed to be in contact.

### Predicting residue contacts

Using the three contact definitions, all residue pairs from different TM helices were labelled as contacting or non-contacting, resulting in a substantial bias of approximately 1∶50. In order to balance training sets and reduce learning time, non-contacting examples were selected randomly in order to achieve approximately equal numbers of positive and negative examples, before fine adjustment of the SVM cost-factor parameter achieved a 1∶1 ratio.

SVM input features were based largely on PSI-BLAST profile data, generated as described above. We used a sliding window of 7 residues, centred on each residue in the pair to produce a feature vector of length 280. Again, this window size was determined by randomly splitting the data set. In addition to profile data, the raw SVM scores for predicted lipid exposure were added to the feature vector for each residue. We then added a number of sequence derived statistics. To define the sequence separation between the two residues, a binary vector was used corresponding to distances of 50, 75, 100, 125, 150, 175, 200 and greater than 200 residues. We also added a value which corresponded to the relative position of each residue within the two TM helices, generated by dividing the residue position in the TM helix by the helix length, and subtracting the value from one where the two residues were on adjacent TM helices or are separated by an even number. This value effectively represented a relative Z-coordinate for each residue, the rationale being that residues separated by a large degree on the Z-axis were unlikely to contact. We tried adding a number of additional values including the lengths of each TM helix, average lipid exposure scores for each TM helix, total number of TM helices, sequence length, and a number of residue co-evolution scores [34],[35]. However, none of these values increased classification performance so were removed in the final model. Again, each feature position was normalised by Z-score, before the target sequence and any other sequences with an E-value less than 1e-4 were excluded from training sets. A radial basis function kernel was used and MCC was used to optimise SVM parameters.

### Using helix-helix prediction for discriminating decoy helical packing arrangements

We then tested the ability of the method to discriminate native from decoy helical packing arrangement using the predicted helix-helix interactions. For each of the 74 multi-spanning proteins in our data set, decoys were generated using the REVCAS program [36]. Each chain was expanded into a larger set of structures by making it circular and introducing cyclically permuted breaks. The method involves a triple-point chain reconnection that avoids the restoration of native segments allowing the generation of a set of decoy structures. The method was successfully applied to the pore-forming colicin domain, an all alpha-helical structure that is typical of many TM proteins in that the amino and carboxy termini, which are joined when the structure is circularised, are at opposite ends of the protein, much like TM proteins whose termini are on opposite sides of the membrane [36]. By generating decoys in both forward and reverse directions, 24–48 decoys were generated for each protein resulting in a total set of 2811 structures. Decoys only contained C-alpha atoms, therefore the remaining backbone and side chain atoms were added and the structure was refined and energy minimised using the Jackal package [37]. Additionally, homology models of the native structures were constructed using MODELLER [38]. Native topologies were then used to define TM helix boundaries allowing observed helix-helix interactions to be extracted which were then compared to the helix-helix interactions predicted from sequence. Decoys and native structures were then scored by the number of interacting/non-interacting helices that matched the predictions and ranked accordingly. We accessed the frequency at which the native structure, or a model of the native structure, was ranked first.

### Constructing the helical packing arrangement

Once helix-helix interactions have been predicted, the helical packing arrangement is treated as an undirected graph where the helices form vertices and their interactions form edges. A force-directed algorithm is then applied which treats the graph as a virtual physical system. The system is simulated resulting in attractive and repulsive forces being applied to vertices, a process which is repeated iteratively until the system comes to an equilibrium state at which point the final graph layout is constructed.

Using the Boost C++ programming library (http://www.boost.org) we employed a modified version of the Kamada-Kawai force-directed algorithm [39] which generates two-dimensional layouts for connected, undirected graphs. It accomplishes this by treating the graph as a dynamic spring system, where the strength of a spring between two vertices is inversely proportional to the square of the shortest distance between those two vertices, and attempting to minimise the energy within the system. In order to avoid producing a layout with only a local minima, the vertices are first arranged along the vertices of a regular *n*-sided polygon, where *n* is the number of TM helices, via a circular layout function. Given that the number of TM helices in a protein is expected to be less than 30, energy minimisation occurs in a number of seconds on a modern computer, avoiding the high running time typically associated with force-directed algorithms and graphs containing a larger number of vertices. Resulting layouts demonstrate uniform edge length, uniform vertex distribution often showing symmetry, and minimisation of edge crossing – attributes that are common to the arrangement of TM helices and their interactions in native TM protein structures.

In a number of cases, multiple helices share the same interactions resulting in numerous possible arrangements. In all cases where this occurs, a recursive function is used to score each arrangement according to the number of observed same-side loop crossovers. The score is determined by drawing a line (loop) between a pair of helices adjacent in sequence, before incrementing the helix position by two so that comparisons are between lines on the same side. Each line is compared to every other line on the same side and their intersection is established by determining the cross product. This is repeated for each side, before the total number of intersections per side is compared. Particularly when loops are short, it is unusual for loops to cross each other as this may result in side chain clashes. All arrangements are then returned, with those containing the least number of same-side loop crossovers scored highest.

Finally, the constituent residues are superimposed on to their respective TM helices, before a genetic algorithm is used to rotate all helices around their respective Z-axes such that the sum of all predicted residue-residue contact distances is minimised, therefore preventing residues contacts occurring across the longitudinal helix axis. For each TM helix, a value in the range 0-359 is optimised to an accuracy of one degree.

## Results

### Lipid exposure prediction performance

We compared the per residue performance of our lipid exposure predictor to the LIPS method using all TM helix residues from our data set of 77 sequences. The data set contained 336 TM helices composed of 7016 residues of which 3687 were labelled as lipid exposed and 3329 were not, according to CGDB data. Optimal performance was achieved using a radial basis function kernel, a gamma value of 0.6 and a trade-off value of 1.5. The LIPS method produces a per residue score generated by multiplying lipophilicity by positional entropy. The LIPS score that resulted in the optimal per residue performance was found to be 1.56. Using leave-one-out cross-validation, our method achieved a MCC of 0.38 and accuracy of 69.3%, a significant improvement over the LIPS method which scored 0.23 and 61.7% respectively (table 1). Furthermore, the LIPS method is calculated using sequence profiles from 18 TM protein structures, the majority of which are included in the test set of 77, therefore in the absence of cross-validation these results are likely to be an overestimate. However, as the LIPS method is based on an alternative definition of lipid exposure, we repeated the benchmarking of the two methods using the LIPS definition by labelling residues with a 1.9 Å probe. Under this definition both methods perform slightly worse although our method still outperforms LIPS, with an MCC value of 0.27 compared to 0.18. This indicates that there is reasonably good correlation between the two definitions although the LIPS definition is slightly harder to predict, most likely because the 1.9 Å spherical probe is a poor approximation to the non-spherical nature of a membrane phospholipid, unlike, for example, a 1.4 Å spherical probe is to a water molecule.

**Table 1:** Per residue lipid exposure prediction performance using a data set of 77 sequences.

Method	Lipid exposure definition	Precision	Recall	FPR	FNR	MCC	Accuracy
MEMPACK	CGDB	0.69	0.56	0.36	0.26	0.38	69.3%
MEMPACK	1.9 Å probe	0.71	0.61	0.39	0.33	0.27	64.3%
LIPS	CGDB	0.61	0.59	0.48	0.29	0.23	61.7%
LIPS	1.9 Å probe	0.65	0.65	0.50	0.32	0.18	60.3%

Lipid exposure definition  =  test set labelled according to the CGDB definition or using a 1.9 Å probe. FPR  =  false positive rate. FNR  =  false negative rate. MCC  =  Matthews Correlation Coefficient. Accuracy  =  (TP + TN)/(TP + TN + FP + FN).

### Residue contact prediction performance

Residue pair contact prediction performance compared with two TM protein contact predictors (TMHcon [16] and TMhit [23]) and two globular protein contact predictors (PROFcon [13] and SVMcon [14]) using the data set of 74 sequences and three contact definitions is shown in table 2. Existing methods all had the option of a *L*5 mode, where only the top *L*/5 positive results are returned where *L* is the sequence length, or for TM protein-specific methods, the total length of all TM helices. This generally has the effect of reducing the false positive rate though usually at the expense of increasing the false negative rate; however our method did not benefit from the use of this scoring method suggesting the SVM hyperplane is already optimally positioned.

**Table 2:** Per residue pair contact prediction performance using a data set of 74 sequences.

Method	Contact Definition	Precision	Recall	FPR	FNR	MCC
MEMPACK	1	0.69	0.0023	0.0010	0.88	0.28
SVMcon	1	0.06	0.00050	0.0083	0.97	0.03
SVMcon L5	1	0.09	0.00	0.0003	1.00	0.01
PROFcon	1	0.03	0.021	0.4600	0.41	0.04
PROFcon L5	1	0.06	0.00010	0.0018	0.99	0.01
MEMPACK	2	0.69	0.0015	0.0007	0.88	0.28
TMhit L5	2	0.57	0.0015	0.0012	0.88	0.26
MEMPACK	3	0.70	0.0022	0.0010	0.89	0.27
TMHcon L5	3	0.09	0.00020	0.0021	0.99	0.02

Contact definition 1 =  A maximal distance of 8 Å between their C-beta atoms (C-alpha for glycine). 2 =  The distance between any two atoms from an interacting pair is less than the sum of their van der Waals radii plus a threshold of 0.6 Å. 3 =  The minimal distance between side chain or backbone heavy atoms in an interacting pair is less than 5.5 Å. Results for contact definition 3 used 58 sequences that had more than 2 TM helices as TMHcon is unable to make predictions for 2 TM helix sequences.

Performance at all three contact definitions was consistent, with a MCC value of approximately 0.28 although a slightly lower false positive rate using contact definition 2. All three SVMs achieved optimal performance using radial basis function kernels with gamma and trade-off values of 24 and 1 respectively. Addition of the predicted lipid exposure scores to profile data in the SVM feature vector resulted in an improvement of approximately 0.05 MCC, while the additional sequence derived statistics contributed approximately 0.03 MCC. Although a combination of residue co-evolution scores did improve performance slightly compared with using profile data alone (0.02 MCC), this increment was lost when scores were added after predicted lipid exposure suggesting the two overlap in feature space.

Compared to existing predictors, our method performed well with MCC scores substantially higher than both SVMcon and PROFcon (contact definition 1) using either standard or L5 scoring schemes. SVMcon L5 was able to produce a lower false positive rate (FPR) but at the expense of a false negative rate (FNR) of 1.0. Similarly, PROFcon produced a lower FNR of 0.41 but at the expense of a higher FPR of 0.46, compared to 0.001 for our method. On this evidence, globular protein contact predictors appear to perform relatively poorly when applied to TM proteins. In comparison to TMhit, a recent SVM-based TM protein contact predictor, results were more comparable. While our method scores higher on all assessment metrics, the margin of improvement is narrower with a MCC of 0.28 compared to the TMhit value of 0.26. This is not unexpected given that both methods use SVM classifiers, though more significantly there is a considerable overlap of 42 sequences in training sets. Given that we assessed our method using leave-one-out cross-validation whereas TMhit results were not cross-validated, TMhit results are likely to be overestimated therefore the actual margin of improvement may be larger. Compared to TMHcon, a recent neural network based approach, our method again performed well, with TMHcon results comparable to the globular protein contact predictors.

### Helix-helix interaction prediction performance

We assessed performance of helix-helix interaction prediction requiring one residue from each helix to be in contact. Based on observed interactions there were comparable numbers of interacting and non-interacting helices for all contact definitions, with 668 and 733 respectively using contact definition 1. Results using the data set of 74 sequences and three contact definitions is shown in table 3.

**Table 3:** Helix-helix interaction prediction performance using a data set of 74 sequences.

Method	Contact Definition	Precision	Recall	FPR	FNR	MCC	Accuracy
MEMPACK	1	0.93	0.10	0.0087	0.84	0.29	64.7%
SVMcon	1	0.57	0.11	0.090	0.84	0.11	59.3%
SVMcon L5	1	0.82	0.034	0.0074	0.95	0.13	59.5%
PROFcon	1	0.43	0.16	0.83	0.16	0.02	45.4%
PROFcon L5	1	0.72	0.11	0.043	0.84	0.19	62.0%
MEMPACK	2	0.95	0.11	0.0062	0.84	0.29	63.6%
TMhit L5	2	0.77	0.31	0.12	0.47	0.45	73.2%
MEMPACK	3	0.94	0.11	0.008	0.85	0.27	60.6%
TMHcon L5	3	0.49	0.32	0.37	0.63	0.02	52.3%

Successful prediction of interacting helices requires one residue from each helix to be in contact. Results for contact definition 3 used 58 sequences that had more than 2 TM helices as TMHcon is unable to make predictions for 2 TM helix sequences.

Our method achieved similar scores using contact definitions 1 and 2, with a MCC of 0.29 and accuracies of 64.7% and 63.6%. Using contact definition 3, results were slightly lower with a MCC of 0.37 and accuracy of 60.6%. The FNR was consistent across all definitions at approximately 0.84. Compared to SVMcon and PROFcon, our method performed well with only PROFcon L5 approaching similar performance (MCC 0.19, accuracy 62.0%), suffering only from a higher FPR compared to our method. Other than PROFcon L5 which performed better than expected for a globular protein predictor, results were generally low with MCC values in the range 0.02–0.13. The performance of TMhit surpasses that of our method with MCC 0.45 and accuracy 72.3%. However, as described above, the TMhit results were not cross-validated and are likely to be substantially overestimated given the overlap of 42 sequences in training sets. To give an estimate of the level of improvement this is likely to have resulted in, we scored our method in the absence of cross-validation for the 42 overlapping sequences and achieved scores of MCC 0.65 and accuracy 82.6%. We additionally compared the two methods using a smaller data set of 14 sequences for which both our method and TMhit results were fully cross-validated [23]. Requiring a single contacting pair of residues, our method achieved 66.3% accuracy compared to 39.1% for TMhit (standard error ±5%). TMHcon achieved MCC 0.02 and accuracy of 52.3%, which reflected the relatively poor performance in residue contact prediction, caused largely by a high FPR of 0.37.

### Helical packing arrangement decoy discrimination performance

Using our decoy set, we were able to derive between 1 and 53 (average 18.5) unique helical packing arrangements for 71 sequences in our data set. By combining these with unique helical packing arrangements derived from the native crystal structure and homology models of the native crystal structure, we assessed performance of our and existing methods at discriminating the native or native model arrangements from decoy arrangements. Each arrangement was scored according to the number of interacting/non-interacting helices that matched the prediction from sequence, with interacting/non-interacting helices scored equally. Accuracy was determined by counting the frequency at which the native or native model arrangement achieved the highest score. As discriminating 2 TM helix arrangements, where helices are either interacting or not, is somewhat trivial, table 4 shows results including and excluding 2 TM helix arrangements, where there are a total of 57 sequences with more than 1 unique packing arrangement.

**Table 4:** Helical packing arrangement decoy discrimination using a data set of 71 sequences with 2 or more TM helices (n = 71) and a data set of 57 sequences with 3 or more helices (n = 57).

Method	Contact Definition	Accuracy (n = 57)	Accuracy (n = 71)
MEMPACK	1	68.4%	69.0%
SVMcon L5	1	52.6%	56.3%
PROFcon L5	1	45.6%	52.1%
MEMPACK	2	66.6%	67.6%
TMhit L5	2	59.6%	66.2%
MEMPACK	3	70.2%	70.4%
TMHcon L5	3	40.4%	-

Accuracy reflects the frequency at which the native or native model helical packing arrangement achieved the highest score compared to the decoy set.

Consistent with prediction of helix-helix interactions, our method performed similarly using contact definitions 1 and 2, although unexpectedly performed best using contact definition 3 (70.4% accuracy). Excluding 2 TM helix proteins, using all contact definitions, performance decreased slightly suggesting that, on average, discriminating 2 TM helix arrangements is slightly easier than for other topologies. SVMcon and PROFcon both performed best when evaluated using their L5 modes although both achieved accuracies over 10% lower than our method. TMhit achieved a slightly lower score than our method (66.2%) though again in the absence of cross-validation. Excluding 2 TM helix proteins performance was almost 7% lower. TMHcon was not assessed using the complete set of 71 as it is unable to make predictions on 2 TM helix proteins, and performed below all other methods (40.4% accuracy) on the set of 57.

### Assessing the accuracy of helical packing arrangements

Given that the generation of helical packing arrangements is based on the interconnection of vertices within a graph, accuracy is ultimately dependent on the detection of edges via prediction of helix-helix interactions. Out of the data set of 74 sequences, 17 (23%) had all interactions successfully predicted although in 3 of these cases there were no observed interactions between helices. Predicted arrangements were then compared by visual inspection of a two-dimensional slice taken from the crystal structure approximately normal to the likely plane of the lipid bilayer, and assessed based on the overlap of helices from the predicted arrangement and the slice. Of these 17 cases, 9 arrangements produce overlaps for all TM helices and therefore can be considered as closely resembling the helix packing arrangement observed in the crystal structure.

**Figure 1:**
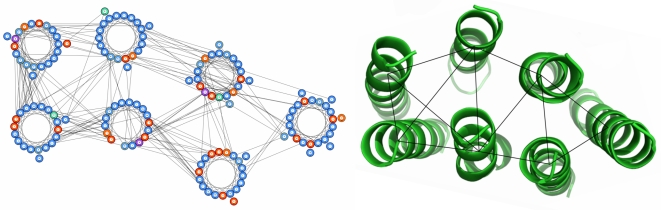
Predicted helical packing arrangement and crystal structure of Halorhodopsin (1E12:A). In this example the two left-most helices share the same interactions. The correct arrangement has been identified as having no same-side loop crossovers, compared to one for the incorrect arrangement. Predicted residue-residue contacts are annotated on the packing arrangement while observed helix-helix interactions are annotated on the crystal structure.

Among these 9 correct cases, three 7 TM helix proteins (PDB: 1E12:A, 1XIO:A, 2F95:A) produced helical packing arrangements that clearly resembled their respective crystal structures (Figure 1). Additionally, for each of these cases the correct arrangement was successfully determined from alternatives by scoring arrangements based on the number of same-side loop crossovers. Overall, this function successfully identified the correct arrangement in 4 out of 6 cases where multiple arrangements were generated when tested using observed helix-helix interaction information; in the remaining 3 cases, 2 had an equal number of crossovers for each of the alternative arrangements (2HYD:A, 1XFH:A) – in these instances, the highest scoring arrangement was the one with the lowest total residue-residue contact distance resulting in one correct and one incorrect prediction, while in the remaining case the correct arrangement contained one more crossover than the incorrect arrangement (1XME:A).

**Figure 2:**
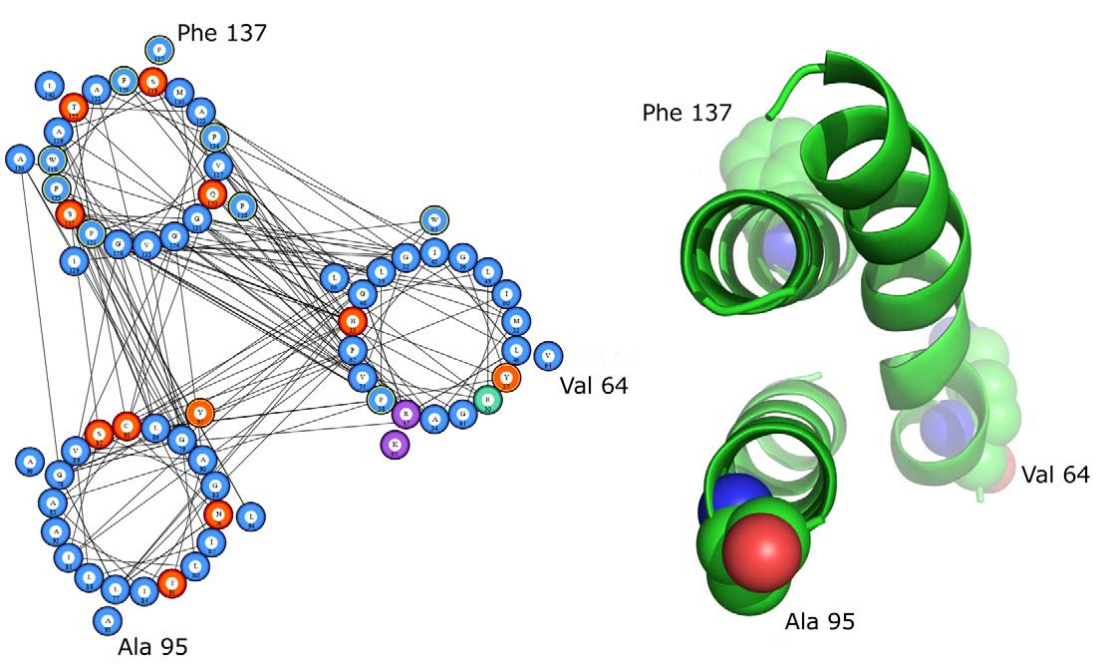
Predicted helical packing arrangement and crystal structure of Photosystem I chain D (1JB0:L). Application of a genetic algorithm to rotate helices about their Z-axes results in the correct positioning of residues Val64, Ala135 and Phe137.

Other cases where all helix-helix interactions were successfully predicted and packing arrangements closely resembled crystal structures included the 5 TM helix ubiquinol oxidase (1FFT:C) and 6 TM helix Aquaporin-4 (2D57:A). Below 4 TM helices, arrangements generally resembled crystal structures well although the task becomes more straightforward as the number of TM helices decreases. Where all helix-helix interactions were successfully predicted and packing arrangement resembled the crystal structure, application of a genetic algorithm to rotate helices around thei respective Z-axes usually resulted in helix orientations that aligned significantly better with native structures compared to arbitrary degrees of rotation (Figure 2).

When helices were connected consecutively, for example where a 3 helix protein has interactions between helices 1–2 and 2–3, the program was unable to determine the correct arrangement despite predicting all helix-helix interactions correctly. Under these circumstances, the algorithm defaults to a circular layout, which is frequently closest to the crystal structure as in the case of aquaporin (2D57:A) where helices are arranged around a central pore. In a number of cases though, the correct arrangement is much closer to linear as in the case of Photosystem II (2AXT:A) where there is significant interaction with additional chains in the complex. In such situations, the helix-helix interactions alone do not provide enough information to determine the correct arrangement.

Where prediction of helix-helix interactions falls below 100%, packing arrangements generally fail to accurately resemble crystal structures. In some cases such as the ammonium transporter (2B2F:A), well connected sub-components of 3–5 TM helices were often correctly formed, but their arrangement in relation to each other was incorrect due to a number of missing helix-helix interaction. In three cases where there was substantial interconnection between TM helices, the arrangement does not succeed, most likely due to the algorithm encountering a local minima. It is also impossible to generate an arrangement from a disconnected graph, where all helix-helix interactions are incorrectly predicted, which occurs in 12 sequences (16.2%). A summary of results where all interactions were correctly predicted is shown in Table 5.

**Table 5:** Assessment of predicted helical packing arrangements for the 17 sequences where all interactions were successfully predicted.

Helical packing arrangement prediction	Count
Resembles two-dimensional slice from crystal structure	9
No observed helix-helix interactions	3
Incorrect due to linear configuration	3
Incorrect helix placement	2

Arrangements were compared to a two-dimensional slice taken from the respective crystal structures and assessed based on the alignment between the helices in the predicted arrangement and in the slice; in 9 cases there was overlap for all helices (2F95:A, 1E12:A, 1XIO:A, 2D57:A, 1FFT:C, 1JB0:L, 1C17:A, 1R3J:C, 2AHY:A). In 3 cases, there were no observed helix-helix interactions therefore no arrangement could be predicted (1VCR:A, 1YQ3:D, 1ZOY:C). In 3 cases, the arrangement predicted a circular configuration whereas the correct arrangement was approximately linear (1DXR:M, 2AXT:D, 2AXT:A).

**Figure 3:**
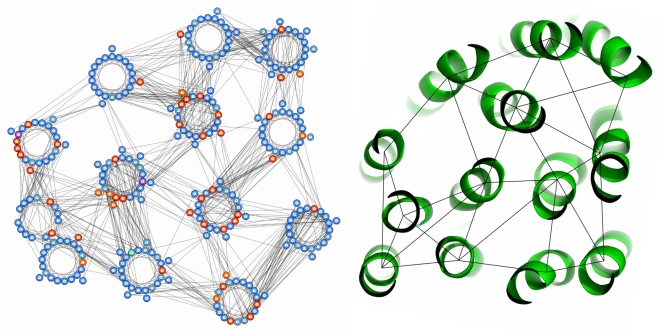
Helical packing arrangement and crystal structure of cytochrome C oxidase (1XME:A), generated using observed rather than predicted helix-helix interactions. Observed residue-residue contacts are annotated on the packing arrangement while observed helix-helix interactions are annotated on the crystal structure. In this example, the two helices at the bottom left of the arrangement are incorrectly placed; they share the same helix-helix interactions but the correct arrangement has one same-side loop crossover whereas the incorrect arrangement has none. The alternative correct arrangement where the placement of these two helices is reversed is returned as the second highest scoring arrangement.

While the successful packing arrangements were achieved with topologies of less than 8 TM helices, we additionally tested the algorithm using observed data to validate its effectiveness at generating arrangements for topologies with large numbers of TM helices using observed helix-helix interaction data rather than predicted contacts. In a number of cases, complex packing arrangements were generated with up to 13 TM helices that clearly resembled the respective crystal structure. Examples include the 10 TM helix proton ATPase (1MHS), 12 TM helix multidrug transporter (2GFP:A) and 13 TM helix cytochrome C oxidase (1XME:A) shown in figure 3, although in this case two helices that share the same helix-helix interactions are incorrectly replaced.

## Discussion

In this paper we have implemented a novel tool capable of predicting lipid exposure, residue contacts and helix-helix interactions using SVM classifiers. These predictions are then combined to produce the optimal helical packing arrangement using a force-directed algorithm. Firstly, lipid exposure is predicted using evolutionary information labelled by data derived from coarse-grained molecular dynamics simulations. Solvent-exposed residues in both globular and TM proteins are known to be less conserved than buried residues, therefore non-conserved residues are more likely to identify lipid-exposed surfaces of TM helices [40],[41]. But in contrast to globular proteins, TM proteins do not show large differences in hydrophobicity between lipid-exposed and buried residues, making lipid exposure prediction a harder task [42]. Using machine learning tools that have been successfully applied to TM protein topology prediction [4], we were able to achieve per residue accuracy that compares favourably with a recent existing method suggesting the SVM is efficiently capturing the major distinguishing features of lipid exposure, the periodicity of conserved residues and the polarity of their side chains, from sequence profile data. Predictions may be useful for a number of additional applications including the modification of a TM protein-specific energy functions for *ab inito* modelling [9] where they could be incorporated into the potential, as for example ROSETTA [10] includes the LIPS score in its energy function, or added as an additional term with a separate weighting.

By combining predicted lipid exposure with sequence derived statistics and profile data centred on each residue in a pair, we were able to train an additional SVM to predict residue contacts. Recent methods specifically designed to predict residue contacts in TM proteins have used a variety of features including residue co-evolution scores, contact propensities and a range of global sequence-derived values. By experimenting with different combinations we attained optimal performance using a minimal set of features without the need for a consensus approach, resulting in significant improvement compared to all existing methods. Our results demonstrate that globular protein contact predictors perform poorly when applied to TM proteins due to extremely high levels of false negative predictions. This is not especially surprising since the amino acid composition of hydrophobic globular protein alpha-helices has recently been shown to contrast from that of TM helices, therefore contact propensities are likely to differ. Generally, hydrophobic globular protein alpha-helices that are long enough to span the bilayer contain three or more charged residues with a relatively even distribution along their lengths, as well as a decreased frequency of occurrence of Ile and Val residues, while charged residues in TM helices tend to be concentrated towards helix termini [43]. Additionally, in the case of PROFcon, all TM proteins were removed from the data set so the neural network had received no training with TM protein data. Compared to the top performing TM protein contact predictor, our method achieves higher performance on all assessment metrics despite the lack of cross-validation of TMhit which was trained on a data set which included 42 sequences that are present in our test set. While our method produces a consistently low FPR, the FNR achieved a maximum score of 0.89. This result may suggest that our SVM is not sampling feature space effectively, although it is reasonable to suggest that many of these contacts are brought together as a consequence of strongly interacting residues that are correctly predicted. Studies of globular proteins have found that folds could be reconstructed using *ab initio* techniques and distance constraints to obtain native-like structures using between *N*/4 and *N*/8 restraints, where *N* is sequence length [44],[45], which supports the notion that the majority of contacts may be consequential. Ranked by average raw SVM score, the top five predicted contacts include Ala-Ser, Gly-Ile, Ile-Phe, Ala-Trp and Ala-Leu, which is broadly in line with previous observations of a relative enrichment of small and aromatic residues in packing interactions [17],[18],[46]. Residue contacts involving a pair of charged residues occur in between 16 and 20 of the 74 proteins (depending on contacting definition), with most containing only a single charged pair. Therefore they are relatively under-represented in the current data set. Out of 53 contacting charged pairs across all contact definitions, only 10 are correct so compared to uncharged contacts they are poorly predicted by the SVM. Aside from a relative lack of training data, it is difficult to speculate on exactly why this is although most are side-chain to backbone interactions. Additional input features may therefore be required to improve prediction of charged residue pairs. However, contacts between some Arg-Asp and Arg-Glu pairs are predicted relatively strongly and are amongst the top 25 scoring predictions.

Helix-helix interaction results generally mirrored contact prediction performance, though globular protein contact predictors faired slightly better due to the relative ease of only having to predict a single residue contact for a successful helix-helix interaction, particularly when the FPR is reduced using the *L*5 mode, with PROFcon achieving 62.0% compared with 64.7% compared to our method. While difficult to compare accuracy using the entire test set of 74 sequences, the significant improvement of our method over TMhit when fully cross-validated on a smaller set of 14 sequences suggests state-of-the-art performance. While it is often difficult to successfully predict all helix-helix interactions correctly, the discrimination of decoy helical packing arrangements provides a measure of how well a method predicts enough interactions correctly to identify the native arrangement, a value which is usually below 100%. Results indicate that our method performs well achieving up to 70.4% accuracy, aided by the fact that 50% of sequences have over 60% of their helix-helix interactions correctly predicted (contact definition 3). PROFcon, achieving only 52.1%, performs much worse than its helix-helix interaction prediction score would suggest, indicating that these successful interaction predictions are limited to a smaller number of sequences, and that prediction generalises poorly across a larger test set, while conversely SVMcon performs better than its interaction prediction score would suggest indicating better generalisation. Again it is difficult to accurately compare TMhit which achieves identical performance.

Using the helix-helix interaction results, helical packing arrangements were constructed using a force-directed algorithm. This task, which was ultimately dependent on the accuracy of predicted interactions, was successful for proteins with up to 7 TM helices although errors occurred where helices were connected consecutively and even correct interaction data was insufficient to identify the correct arrangement. In these circumstances, interactions with additional chains is likely to play a role. For proteins where helix-helix interactions were not all correctly predicted, testing using observed interaction data validated that the algorithm is capable of constructing packing arrangements for proteins with up to 13 TM helices. These results suggested that where predicted helix-helix interactions can be supplemented with interaction data from experimental sources, for example mutagenesis studies, it may be possible to generate accurate packing arrangements for complex proteins containing large numbers of TM helices, assisted by the fast run time of the algorithm that will also allow alternative packing arrangements to be explored iteratively. Predictions can be used to generate pseudo three dimensional-structures with which loop regions can be built using programs such as SuperLooper [47]. Models could then be used to pre-position residues prior to *ab inito* modelling therefore reducing conformational search space and reducing computational requirements.

While our results are encouraging, the paucity of structural data available for training purposes is likely to have limited residue contact and helix-helix interaction prediction performance, particularly as small data sets reduce tolerance to errors and the ability of SVMs to develop large generalisation bounds. Paradoxically, another problem may be the use of crystal structures to derive contact data, which provide only a snapshot of a protein at a given time therefore neglecting the inherent dynamic nature of TM proteins. TM proteins are known to exhibit significant conformational flexibility for a range of functions including modulation of catalytic activity and control of ionic flow, therefore labelling contacts according to a single crystal structure will inevitably lead to training errors. Should enough data become available, it may be preferable to use ensembles of nuclear magnetic resonance structures in place of crystal structures, though due to the experimental difficulties in obtaining membrane protein structures this is unlikely to be an option in the near future. Another issue is the interaction between chains in multimeric complexes, which the majority of TM proteins in structural databases form. It is reasonable to expect that interplay between chains in complexes has a degree of influence on the folding of individual chains, therefore satisfying these oligomeric interactions may lead to an improvement in the fold prediction of individual chains. Predicting oligomeric interactions would also allow TM protein quaternary structure to be predicted from sequence for the first time, while revealing the stoichiometry and symmetry of the complex.

Overall, our results demonstrate that residue contacts and helix-helix interactions can be used to accurately predict the helical packing arrangement of TM proteins, and discriminate native from decoy arrangements. This method can be used to gain insights into TM protein folding, while providing testable hypotheses for a variety of studies including protein design, mutagenesis and thermostability experiments, in addition to reducing conformational search space prior to *ab initio* modelling.

### Availability

MEMPACK is available as source code from the URL below and is free for non-commercial use. All data sets are also available, and cross-validation SVM model files are available on request. The software has been tested on a Linux operating system. In order to compile and run, the gcc compiler, Perl interpreter, Boost C++ libraries and NCBI tools are required. http://bioinf.cs.ucl.ac.uk/memsat/mempack/
